# A thalamic epidermoid cyst presenting with memory disturbances: a case report

**DOI:** 10.1097/MS9.0000000000000557

**Published:** 2023-04-07

**Authors:** Kumar Paudel, Ramesh Shrestha, Sijan Karki, Prabhat Jha, Rajiv Jha

**Affiliations:** a Department of Neurosurgery; b Department of Surgery, National Academy of Medical Sciences (NAMS); c Department of Neurology, Upendra Devkota Memorial National Institute of Neurological and Allied Sciences, Kathmandu, Nepal

**Keywords:** intracranial, memory disturbance, subarachnoid space, thalamus epidermoid cyst

## Abstract

Epidermoid cysts are benign, slow-growing tumors. They account for 0.2–1.8% of all intracranial tumors and rarely appear as intraparenchymal masses. The most prevalent symptom in people of middle age is an insidious-onset headache.

**Case presentation:**

We present a 20-year-old college student who presented with memory disturbances. The imaging revealed a left thalamic mass. The tumor was excised and diagnosed histopathologically as an epidermoid cyst.

**Clinical discussion:**

Epidermoid cysts resemble epidermal skin cells in histology. The lesion of the thalamus involving the ventrolateral and anterior regions is involved with memory and language. Of note, to our knowledge, no cases of memory issues associated with thalamic epidermoid cysts have been reported in the literature.

**Conclusion:**

The ideal treatment is cystic component removal with complete capsule excision. Sometimes, in cases of incomplete excision, radiotherapy can be another option.

## Introduction

Epidermoid cysts are slow-growing benign tumors^1^. Intracranial epidermoid cysts account for 0.2–1.8% of all intracranial tumors, and although they can occur as supratentorial, intraparenchymal masses, they are relatively rare in this location^1^,^2^. The etiology of intraparenchymal epidermoid cysts is unknown^3^. However, the primary cause is gastrulation dysembryogenesis, with secondary disruption of neural tube closure occurring between the third and fifth weeks of gestation^4^. Intradural epidermoid cysts are usually found in the subarachnoid space, insinuating themselves among adjacent structures^5^. They may have varied presentations depending on the intracranial location. In middle-aged patients, the most common presentation is an insidious-onset headache^6^. We are reporting the case of a 20-year-old student who presented with memory disturbances without any focal neurological defect in the background of the thalamic epidermoid. Computed tomography (CT) scans revealed a hypointense mass centered in the left thalamus. We are presenting the case of a patient according to the SCARE (Surgical CAse REport) guideline by Agha *et al*.^7^, where we reviewed the clinical presentation, CT imaging, operative findings, and histopathological pictures.

## Case summary

**Figure 1:**
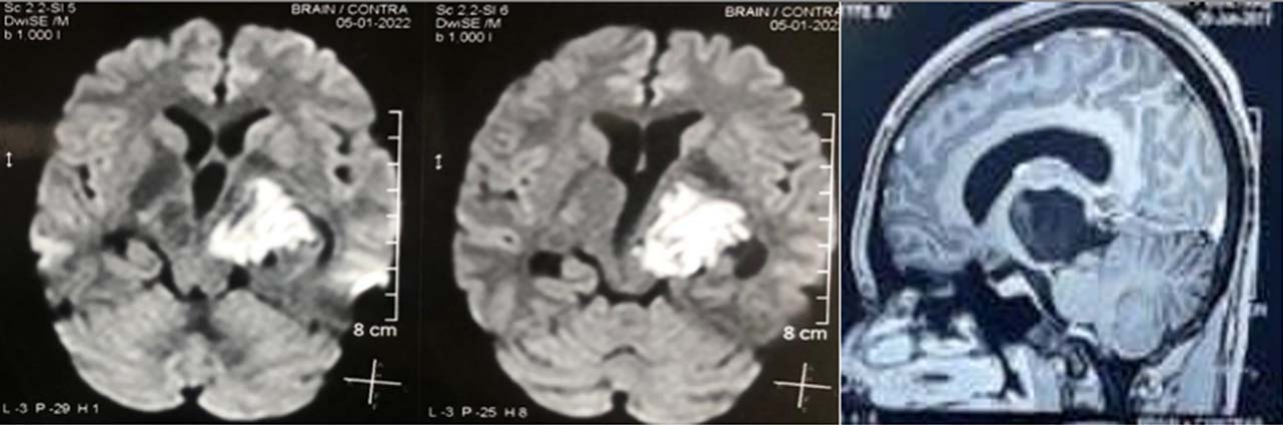
Left thalamic mass.

We have a 20-year-old college student who presented to our facility after experiencing memory problems for 3 months. He underwent a neuropsychological assessment set of cognitive tests – attention, social cognition, language, visual perception, and the Montreal Cognitive Assessment (MoCA). Except for a MoCA of 26/30 and a mild attention deficit, the patient had no focal neurological deficits on the neurological examination. The CBC (complete blood count), CMP (comprehensive metabolic panel), TSH (thyroid stimulating hormone), and vitamin B12 were all normal, and the CSF-PCR (cerebrospinal fluid-polymerase chain reaction) viral panel, RPR (rapid plasma reagin) and HIV (human immunodeficiency virus) were negative. A head MRI revealed a hypointense tumor in the left thalamus measuring about 4 cm and was hypointense on T1-weighted imaging with marked diffusion restriction, distinguishing it from an arachnoid cyst (Fig. 1).

Initially, the lesion was approached through a left pterional craniotomy. The tumor and its capsule were excised. Specimens were collected for the biopsy. The patient awoke at his preoperative neurological baseline.

**Figure 2:**
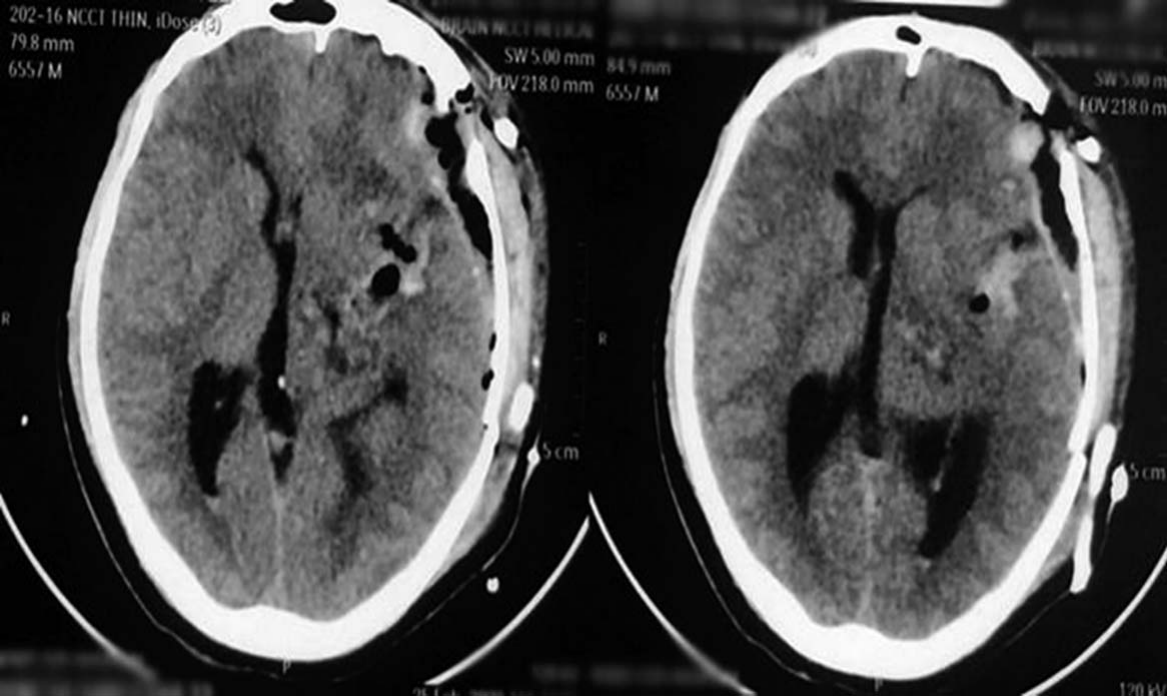
The postoperative noncontrast computed tomography images showing the total excision of the lesion.

Postoperative CT head demonstrated gross total resection of the lesion with preservation of the thalamus (Fig. 2). The intraparenchymal or subdural air-attenuating regions and the calvarial defect are immediate postoperative changes. The effacement of the body, frontal, and occipital horns of the left lateral ventricles is likely due to immediate postoperative brain parenchymal edema.

**Figure 3:**
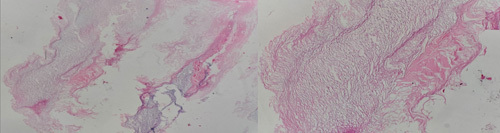
Histological finding of an epidermoid cyst.

The histological specimen revealed keratin flakes, which constitute the cyst’s content (Fig. 3). The lining of the cyst wall was composed of stratified squamous epithelium with a granular layer, a characteristic of epidermoid, and the diagnosis was confirmed. The patient was discharged in 2 days without any postoperative complications. He is in regular follow-up for neurosurgery and neuropsychological evaluation with improvement in memory and neurological function

## Discussion

Intracranial epidermoid cysts are slow growing and account for 0.2–1.8% of all intracranial tumors^8^. The cause is primarily a gastrulation dysembryogenesis, with secondary disruption of neural tube closure, during the third to the fifth week of gestation with growth patterns similar to the epidermal cells of the skin^9^–^12^. Intracranial epidermoids are a rare disorder seldom reported in the literature.

Intradural epidermoids typically infiltrate among nearby structures in the subarachnoid space. Depending on their location inside the brain, they may manifest in different ways. Only a small percentage of people with this condition present in childhood since the tumor grows so slowly, but insidious headaches that start in middle age are the most typical symptom^13^. Common sites for subdural or intradural epidermoid include the cerebellopontine, suprasellar, and cerebellum. In 1957, Lepoire and Pertuiset^14^ described a system based on the embryonic relation for classifying epidermoid that included three groups based on the main blood vessels at the base of the epidermoid (vertebrobasilar, carotid, and choroidal arteries), which are retrosellar, suprasellar, and intraventricular, respectively. In addition to the typical presentation of headache, intracranial epidermoid can develop primary optic atrophy, hemianopia, diplopia, nystagmus, oculomotor disorders, epileptic episodes, hemiparesis, speech problems, dysarthria, and gait disturbance^15^. This wide range of symptoms shows that numerous neurovascular bundles, ranging from the medulla to the optic chiasma, may be involved^16^.

The intriguing aspect is that, to our knowledge, no memory issues have yet been documented in the literature. Moreover, one of the reasons why cerebral epidermoid symptoms are distinctively heterogeneous is because of the several locations where they can develop.

The functional interaction between the ventrolateral and anterior regions of the thalamus contributes to widespread damage of attention circuits between the superior frontal cortex and prefrontal cortex, as well as memory circuits (between the hippocampus and posterior cingulate). In addition, Vilkki’s^19^ study also suggests that the ventrolateral thalamus is involved with memory and language. Hence, we put forward the hypothesis that the thalamic epidermoid cyst has affected the above-mentioned region and tract, which has impaired our patient’s memory function.

The preferred treatment for intracranial space-occupying lesions is the definitive surgical removal of the tumor^20^. However, neurosurgeons must exercise caution when excising the cyst to avoid spillage of its contents into the subarachnoid space, which can increase the patient’s risk of reactive meningitis^21^. If necessary, the dermal tract and cystic extension should be removed, although difficult anatomic locations may pose a challenge^22^. When total excision is not possible, a precise diagnosis from a biopsy can be used to determine whether radiotherapy is a viable option, as germinomas are generally radiosensitive^22^.

## Summary

Histologically, epidermoid cysts resemble epidermal skin cells and are slow-growing tumors. The cause is primarily gastrulation dysembryogenesis, with secondary disruption of neural tube closure. A headache is a common ailment that the majority of people experience. However, occasionally it manifests with peculiar symptoms, as in our case. The best course of treatment is total excision of the capsule and removal of all cystic components, as we performed in our case. Radiotherapy is occasionally a different alternative in circumstances of incomplete excision.

## Ethical approval

The IRC granted ethical approval.

## Patient consent

Written informed consent was obtained from the patient for the publication of this case report and accompanying images. A copy of the written consent is available for review by the Editor-in-Chief of this journal on request.

## Sources of funding

No financial or material support was received for this research or the creation of this work.

## Author contribution

K.P.: contributed to the study concept, data collection, interpretation, and manuscript outlining; R.S.: contributed to the study concept, data interpretation, and critical revision of the manuscript for content; S.K., P.J., and R.J.: contributed to the study concept and critical revision of content. All named authors accept overall responsibility for the work’s integrity and have given their final approval for its publication.

## Conflicts of interest disclosure

None of the authors has any relevant financial or nonfinancial relationships to disclose, as defined above.

## Guarantor

Kumar Paudel.

## Research registration unique identifying number (UIN)

1. Name of the registry: not applicable.
2. Unique identifying number or registration ID: not applicable.
3. Hyperlink to your specific registration (must be publicly accessible and will be checked): not applicable.

## Provenance and peer review

Not commissioned, externally peer-reviewed.
